# Early prenatal exposure to air pollutants and congenital heart disease: a nested case-control study

**DOI:** 10.1265/ehpm.22-00138

**Published:** 2023-01-13

**Authors:** Zhao Ma, Weiqin Li, Jicui Yang, Yijuan Qiao, Xue Cao, Han Ge, Yue Wang, Hongyan Liu, Naijun Tang, Xueli Yang, Junhong Leng

**Affiliations:** 1 Department of Occupational and Environmental Health, School of Public Health, Tianjin Medical University, Tianjin, China; 2 Tianjin Key Laboratory of Environment, Nutrition and Public Health, Tianjin, China; 3 Center for International Collaborative Research on Environment, Nutrition and Public Health, Tianjin, China; 4 Tianjin Women and Children’s Health Center, Tianjin, China

**Keywords:** Air pollution, Congenital heart disease, Gestational exposure, Nested case-control study, Lag effect, Exposure windows

## Abstract

**Background:**

Congenital heart disease (CHD) is one of the most common congenital malformations in humans. Inconsistent results emerged in the existed studies on associations between air pollution and congenital heart disease. The purpose of this study was to evaluate the association of gestational exposure to air pollutants with congenital heart disease, and to explore the critical exposure windows for congenital heart disease.

**Methods:**

The nested case-control study collected birth records and the following health data in Tianjin Women and Children’s Health Center, China. All of the cases of congenital heart disease from 2013 to 2015 were selected matching five healthy controls for each case. Inverse distance weighting was used to estimate individual exposure based on daily air pollution data. Furthermore, the conditional logistic regression with distributed lag non-linear model was performed to identify the association between gestational exposure to air pollution and congenital heart disease.

**Results:**

A total of 8,748 mother-infant pairs were entered into the analysis, of which 1,458 infants suffered from congenital heart disease. For each 10 µg/m^3^ increase of gestational exposure to PM_2.5_, the ORs (95% confidence interval, 95%CI) ranged from 1.008 (1.001–1.016) to 1.013 (1.001–1.024) during the 1^st^–2^nd^ gestation weeks. Similar weak but increased risks of congenital heart disease were associated with O_3_ exposure during the 1^st^ week and SO_2_ exposure during 6^th^–7^th^ weeks in the first trimester, while no significant findings for other air pollutants.

**Conclusions:**

This study highlighted that gestational exposure to PM_2.5_, O_3_, and SO_2_ had lag effects on congenital heart disease. Our results support potential benefits for pregnancy women to the mitigation of air pollution exposure in the early stage, especially when a critical exposure time window of air pollutants may precede heart development.

**Supplementary information:**

The online version contains supplementary material available at https://doi.org/10.1265/ehpm.22-00138.

## Graphical Abstract

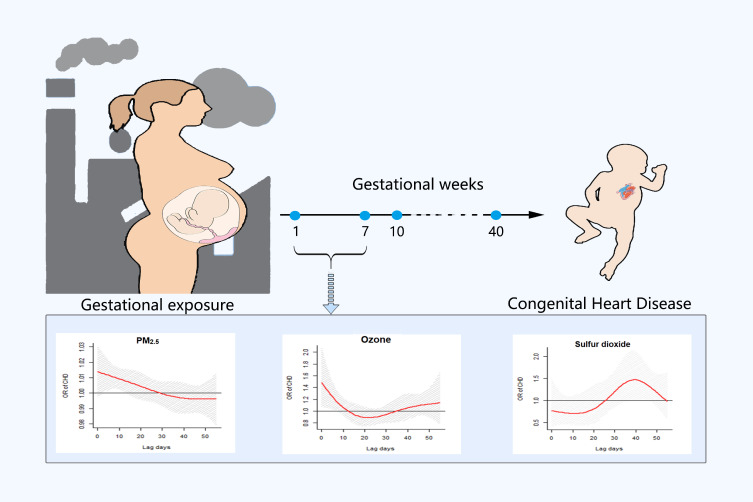

## 1. Introduction

Congenital heart disease (CHD) is one of the most common congenital malformations in humans, affecting nearly 1% of births [1–3]. The global prevalence of CHD at birth is estimated to be nearly 18 cases per 1000 live births in 2017, a 4.2% increase since 1990 [4]. As the world’s most populous country, total CHD prevalence in China increased from 0.2‰ to 4.9‰ in 1980–2019 [5]. Despite a strong heritable basis, the genetic etiology is identified in less than 20% of CHD cases [6, 7]. The development of CHD is multifactorial with genetic and environmental influences [8–10]. Although two systematic reviews indicated that increased risk of CHD was associated with gestational air pollution exposure [11, 12], a few studies did not observe the effects of air pollution on CHD during pregnancy [13, 14]. Furthermore, the current evidence is mainly derived from European and American countries with lower air pollution levels [15–17]. The association studies were still limited, especially in areas with relatively high concentrations of pollutants.

In addition, maternal exposure to air pollution in the early stages of pregnancy may harm the development of the fetal heart since the sensitive period of organ formation was 3–8 weeks after fertilization in gestation [18]. Several epidemiological studies have suggested that gestational exposure to particulate matter and/or gaseous pollutants can increase the risk of CHDs during the first trimester [19–21]. However, a few studies found that the susceptibility windows of air pollutant were mainly in the second and third trimesters of pregnancy [22]. Potential sensitive exposure window of air pollution linking to risk of CHD remained inconsistent in the early stage of pregnancy.

Therefore, we conducted a nested case-control study in Tianjin, a city located in northern China, to assess impact of air pollutants on CHD during the early stage of pregnancy and to explore the critical exposure window for CHD.

## 2. Methods

### 2.1 Case-control selection

We performed a nested case-control study to explore the delayed effects of gestational air pollution on CHD with a birth cohort data collected from Tianjin Women and Children’s Health Center (TJWCH).

This birth cohort was conducted using a multi-stage randomized cluster sampling, and randomly selected 9 of the 16 administrative regions in Tianjin, which covered the urban and suburb areas. First, pregnant women who resided in these regions with the date of last menstruation period (LMP) from January 1, 2013, through June 31, 2015, were included based on the registration data of TJWCH. Second, maternal information including maternal age, education levels, maternal height, pre-pregnancy weight, gravidity, and parity were obtained from registration data. The birth information of the infants was also collected from TJWCH, including infant sex, birth date, and screening information of CHD. A total of 83,402 mothers were enrolled during 2013–2015 in Tianjin in the birth cohort, among which 1,451 mothers were excluded due to the lack of missing resident address information. Third, we selected all the cases in this cohort and matched them with controls in a 1:5 ratio, according to maternal age (±2 years), the same season of LMP, gravidity, and parity. The Ethics Committee for Clinical Research of Tianjin Women and Children’s Health Center approved the study and analysis plan. Tianjin Women and Children’s Health Center agreed to waive the need for written informed consent from all participants because we used the electronic data set from health care records.

### 2.2 Exposure

The air pollutant data was collected by the database of China National Environmental Monitoring Centre. A 24-hour average concentration was calculated for particulate matter (aerodynamic diameter <10 µm, PM_10_), fine particulate matter (aerodynamic diameter <2.5 µm, PM_2.5_), nitrogen dioxide (NO_2_), sulfur dioxide (SO_2_), and carbon monoxide (CO), whereas an 8-hour maximum average concentration was calculated for ozone (O_3_). We checked residential address information of participant from the health-care records of TJWCH and geocoded it to coordinates. Individual exposure was estimated using the method of Inverse distance weighting (IDW), which calculated the daily pollution weighted by the inverse of the square of distance from the residence address to the air quality monitor. IDW has been applied in several disciplines in environmental science, especially in the study of the association between air pollution and disease [23–25]. Exposure assessment was performed during first trimester period in this study.

### 2.3 Outcomes

TJWCH have launched CHD screening program for all neonates born alive in Tianjin, based on the city-wide coverage of pregnant and children health surveillance system [26]. All children were invited to take part in the CHD screening within three months after births. Cardiac defects were confirmed by color Doppler ultrasonic diagnostic system by clinicians. CHD was defined as a structural abnormality of the heart or great vessels as shown by echocardiography. Ventricular septal defect was defined as having a defect between the left and right ventricles at the interventricular septal level. Atrial septal defect was defined as having a defect and shunt between left and right atria at the interatrial septal level except for patent foramen oval. More details for the echocardiographic standard of other CHD subtypes were published elsewhere [26].

### 2.4 Statistical analysis

Normal distribution data were described by means and standard deviations, and non-normal distribution data were presented by median and interquartile range. Categorical variables were described by frequencies and proportions. We used conditional logistic regression with distributed lag non-linear model (DLNM) to assess the effect of exposure between cases and matched controls. DLNM can simultaneously represent non-linear exposure-response dependencies and delayed effects [27]. The construction of DLNM model mainly includes two steps. First, we need to fit the exposure-response function and the exposure-lag function as basis functions. The exposure-response dimension was built by natural spline function with 3 degrees of freedom. Since CHD was known to occur in the 3^rd^ to 8^th^ weeks of pregnancy [14], we specify the lag days from LMP up to 56 days. A natural spline function was also used for the exposure-lag dimension. Second, we used the two basis functions to establish an exposure-response-lag cross-basis matrix, which included in a model formula of a regression model. Since the study was designed as a nested case-control study (1:5 matching), conditional logistic regression was performed. The conditional logistic regression model was adjusted for maternal education (primary or below, junior high school, high school, and bachelor degree or above), pre-pregnancy body mass index (BMI, continuous variable) and infant sex (boys versus girls) in this study. The odd ratios (ORs) are shown for 10 µg/m^3^ increase in PM_10_, PM_2.5_, NO_2_, SO_2_, and O_3_, while OR for 1 mg/m^3^ increase in CO.

As maternal age is a risk factor for CHD [28], the sensitivity analysis was performed in which we excluded cases aged 35 years old or elder as well as their matched controls to examine the robustness of the results. Besides, previous literature shows that there are sex differences in the relationship between pollutants and congenital heart disease [22]. We conducted a stratified analysis of infant sex to explore the difference relationship between air pollutants and congenital heart disease.

All statistical analyses were conducted by using R version.4.1.1. Package “survival” and “dlnm” were used for the conditional logistic regression and DLNM model, respectively. *P* < 0.05 was used to access the statistical significance.

## 3. Results

### 3.1 Descriptive analysis

**Fig. 1:**
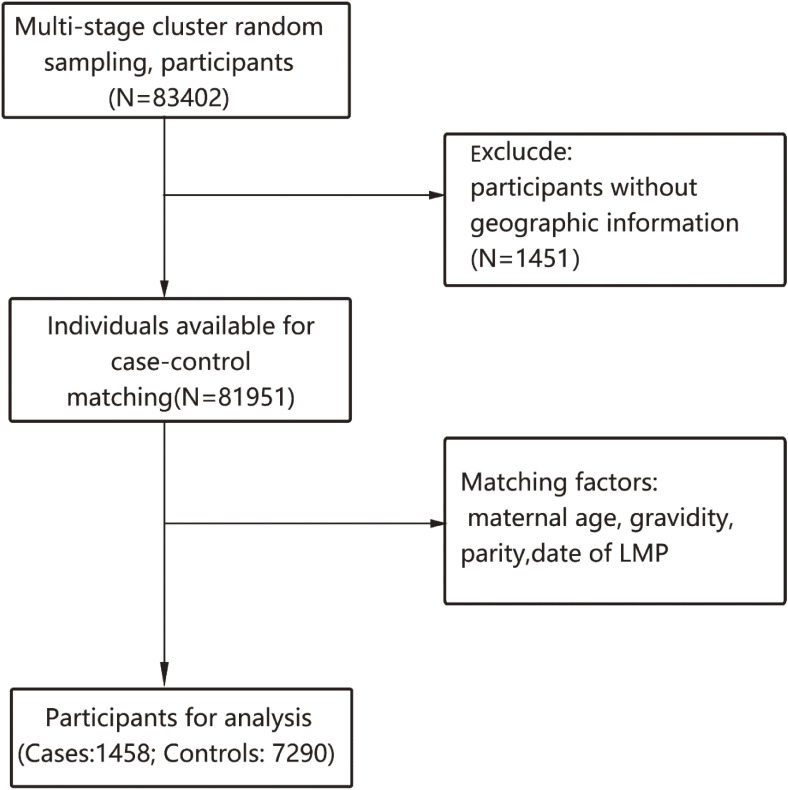
Work flow for case control selection.

**Fig. 2:**
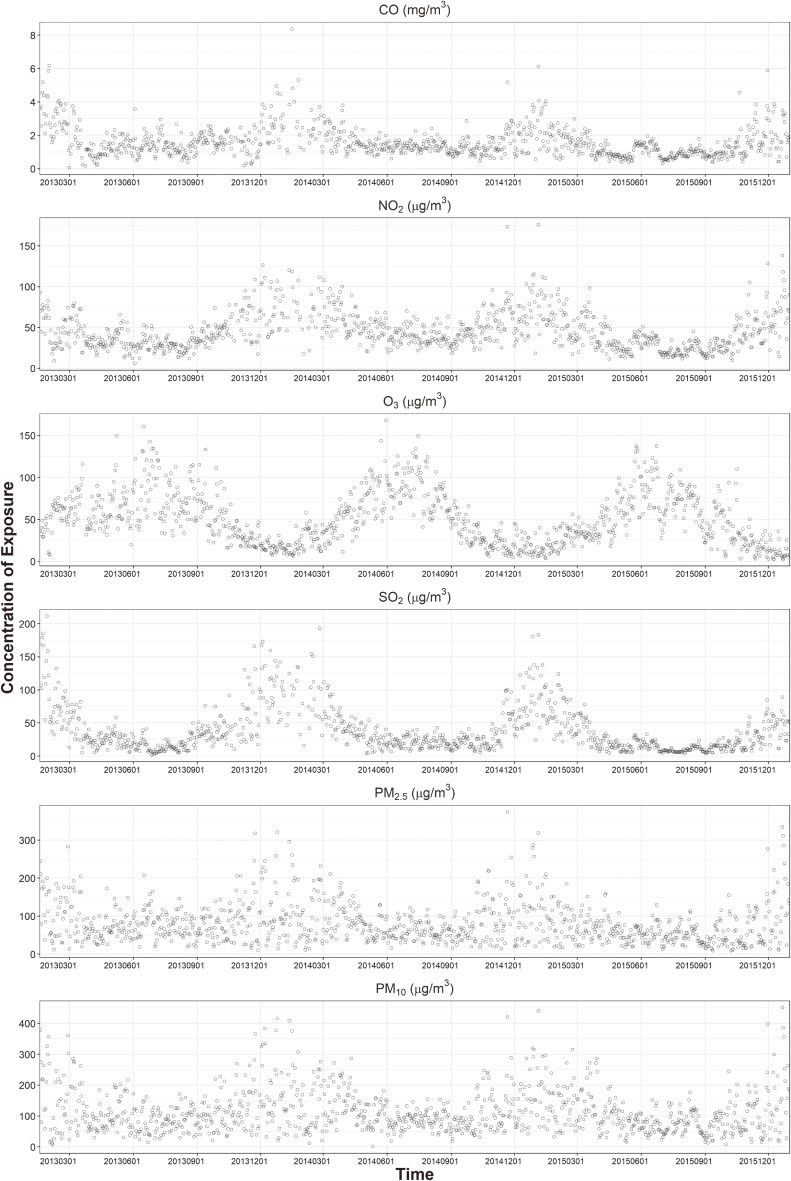
Daily concentration of air pollutants in Tianjin during 2013–2015.

The final analysis included 1,458 CHD cases, matching for 7,290 participants as control. The work flow for the analyzed subjects was displayed in the Fig. 1. The mean maternal age of participants was 28.29 ± 3.68. The average BMI of pre-pregnancy was 22.26 ± 3.73. Table 1 shows the details about characteristics of the study participants. The trend of daily concentration of air pollutants changed from 2013 to 2015 as shown in Fig. 2 and details were shown in eTable 1 in supplementary materials. In general, except for ozone, the concentrations of pollutants were higher in winter and spring than in summer and autumn. Correlations between individual exposure concentration of each pollutant were showed in supplementary material eFigure 1.

**Table 1:** Characteristics of participants (n = 8748).

**Characteristics**	**Cases (n = 1458)**	**Controls (n = 7290)**	**P value**
Maternal age, mean (SD)	28.41 ± 4.22	28.26 ± 3.57	0.546
Pre-pregnancy BMI, mean (SD), kg/m^2^	22.38 ± 3.93	22.23 ± 3.69	0.621
No. (%) with maternal education levels			<0.001
High school or below	969 (66.5)	4469 (61.2)	
Bachelor degree or above	420 (28.8)	2476 (34.0)	
No. (%) with infant sex			<0.001
Boy	615 (42.2)	3755 (51.5)	
Girl	843 (57.8)	3534 (48.5)	

a. The values are presented as percentages (%) or means ± SD.

b. Some columns do not add up to 100% because of missing data.

### 3.2 Regression analysis

**Fig. 3:**
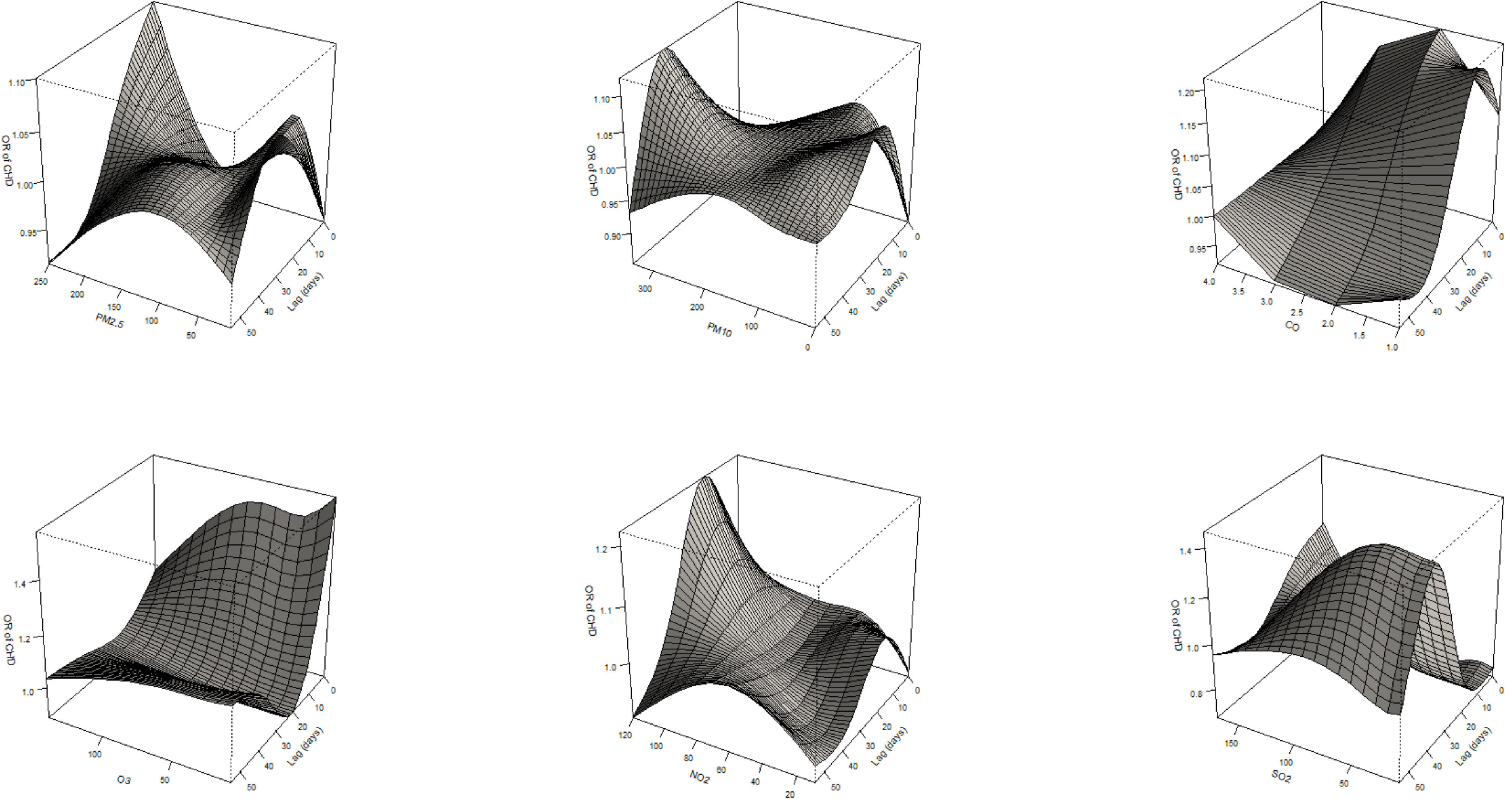
Associations of ORs between air pollutants and CHD. a: 10 µg/m^3^ increase in PM_10_, PM_2.5_, NO_2_, SO_2_, and O_3_; 1 mg/m^3^ increase in CO.

Figure 3 illustrated associations between air pollutants and CHD in DLNM models. Positive associations were observed for the exposures to PM_2.5_, O_3_, SO_2_ in the early stage during pregnancy. For each 10 µg/m^3^ increase of daily gestational exposure to PM_2.5_, the ORs (95% confidence interval, 95%CI) ranged from 1.008 (1.001–1.016) to 1.013 (1.001–1.024). Additionally, the ORs of CHD changed from 1.163 (95%CI: 1.008, 1.342) to 1.485 (95%CI: 1.070, 2.062) for an increase of 10 µg/m^3^ in gestational exposure to O_3_. Positive effects of CHD were also observed with each 10 µg/m^3^ increase of gestational exposure to SO_2_ with ORs ranged from 1.335 (95%CI: 1.014, 1.756) to 1.466 (95%CI: 1.007, 2.136).

**Fig. 4:**
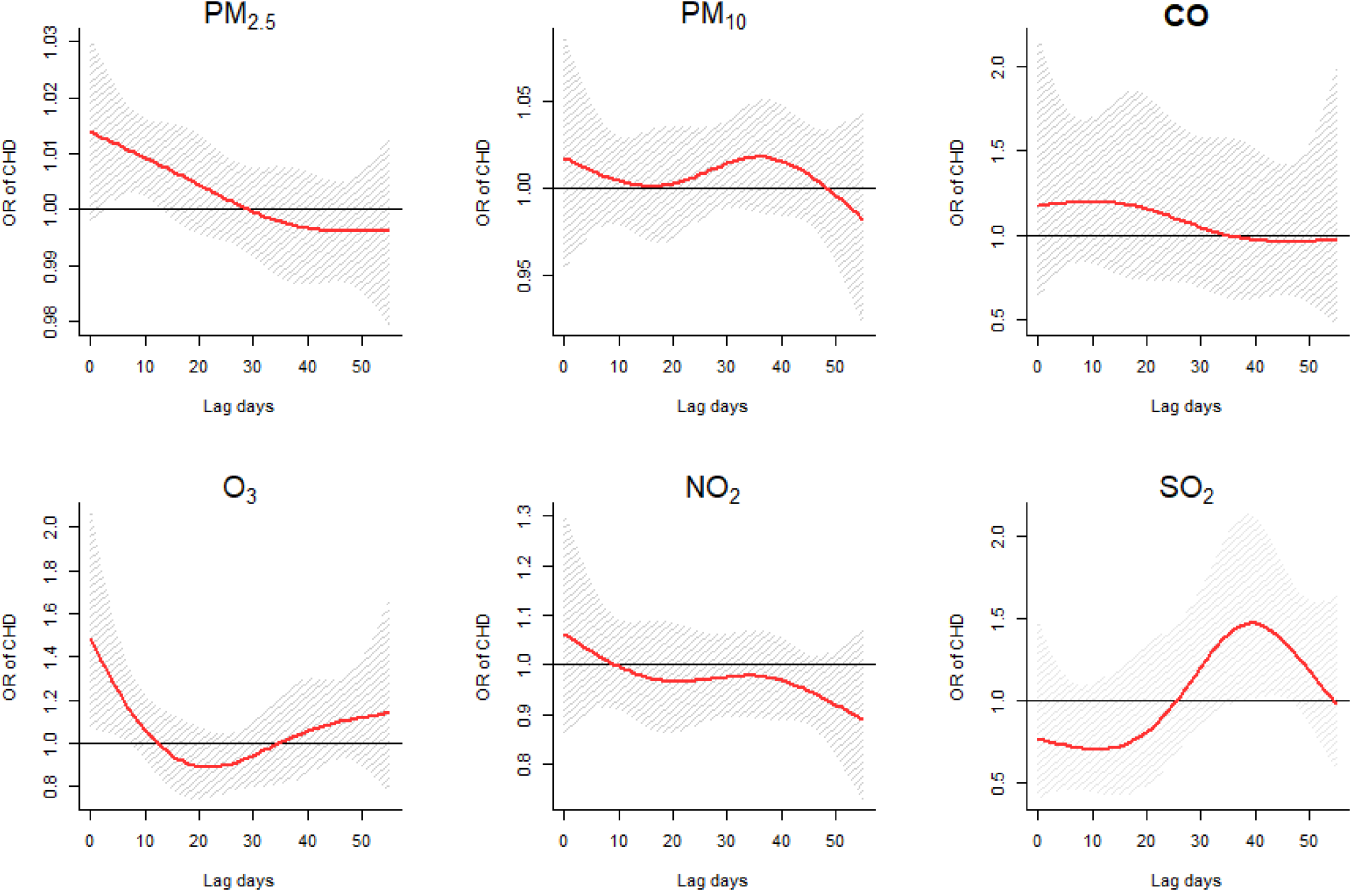
The ORs for different lag days of air pollutants. a: 10 µg/m^3^ increase in PM_10_, PM_2.5_, NO_2_, SO_2_, and O_3_; 1 mg/m^3^ increase in CO.

Figure 4 reflected the ORs for different lag days for effect of each pollutant on CHD. The exposure window varies among different types of pollutants, while it mainly concentrated upon the 1^st^–7^th^ weeks during pregnancy. In our results, vulnerable windows of PM_2.5_, O_3_, and SO_2_ were 1^st^–2^nd^ gestation weeks, the 1^st^ gestation week, and 6^th^–7^th^ gestation weeks, respectively. No association was found in PM_10_, CO, and NO_2_ in our study. The detailed ORs with 95% confidence intervals (95%CI) for CHD associated with each pollutant during the study period were shown in supplementary material eTable 2.

After excluding 140 cases with mother aged 35 years old or elder as well as their matched controls, the ORs for CHD and air pollutants changed slightly, and the results of the sensitivity analysis still supported that air pollutants had different lag times for effect on CHD in the early stage of pregnancy, which was shown in supplementary material eFigure 2–3. In stratified analysis, difference relationships were observed between air pollutants and congenital heart disease in different infant sex. For girls, positive associations were observed between PM_2.5_ and SO_2_, the ORs (95%CI) were ranged from 1.011 (1.001, 1.021) to 1.014 (1.001, 1.026) in 1^st^–2^nd^ gestation weeks, and 1.539 (1.005, 2.357) to 1.658 (1.011, 2.720) in 6^th^–7^th^ gestation weeks, respectively. No association was found between air pollutants and CHD in boys. The results indicated that girl might be more vulnerable in the effects of air pollution on congenital heart disease. Details of the stratified analysis were present in eTable 3.

## 4. Discussion

This nested case-control study indicated that air pollution may have an adverse impact on the risk of CHD. Positive associations were observed between increased risk of CHD and exposure to PM_2.5_, O_3_, and SO_2_ in the early stage during pregnancy. Additionally, the exposure window for the effects of air pollution on CHD varies with different pollutant types, while critical exposure windows of PM_2.5_, O_3_, and SO_2_ were 1^st^–2^nd^ gestation weeks, the 1^st^ gestation week, and 6^th^–7^th^ gestation weeks, respectively. No association was found in PM_10_, CO, and NO_2_.

CHD was one of the most common non-chromosomal anomalies attributed to both genetic and environmental factors [29]. Previous meta-analysis reported that ORs for CHD was elevated by increasing gestational air pollution exposure [11, 30, 31]. For instance, results from national studies in the USA showed that exposure to fine particulate matter was positively associated with CHDs, and ORs varied with the subtype of CHD [32–34]. A case-control study during 2001–2010 in Italy have examined that air pollution may play a critical role in heart development and maternal exposure to the highest daily average values of SO_2_ can increase the risk of CHDs [35]. In addition, Australian study indicated the OR of CHD was 2.96 (95CI%: 1.34, 7.52) for an increase of 5 ppb in gestational exposure to O_3_ [15]. As a matter of fact, most of these studies were performed in developed countries with low levels of air pollution. This nested case-control study was conducted in Tianjin, an industrial city in northern China with high levels of air pollution during 2013–2015. We matched controls for each case and conducted a conditional logistic regression to minify the potential confounding. Increased odd ratios of CHD were observed with higher levels of daily SO_2_ and O_3_ in early stage during pregnancy with ORs ranged from 1.163 to 1.485 and 1.335 to 1.466, respectively. For each 10 µg/m^3^ increase of gestational exposure to PM_2.5_, the ORs (95% confidence interval, 95%CI) ranged from 1.008 (1.001–1.016) to 1.013 (1.001–1.024). The results of sensitivity analysis concurred with preliminary results in this study. Nonetheless, several researches elsewhere did not report significant associations between O_3_, SO_2_, and CHD [36, 37]. The reasons for the differences may be explained by the study populations with different races [38, 39], regional heterogeneity in the mass and compositions of air pollutants, and exposure measurement and CHD subtypes [17, 40].

Additionally, numbers of studies suggested that the first trimester is a vital period for the effects of air pollution on CHD [40–42]. However, inconsistencies and uncertainties remained concerning the critical exposure period. Zhang et al. shows that the susceptibility windows of air pollutants mainly in the second and third trimesters of pregnancy [22]. Another population-based study observed the strongest association for O_3_ was the 24^th^–28^th^ weeks and that for SO_2_ was the 16^th^–21^st^ during the embryology weeks [43]. According to the previous literature, exposure to air pollutants is associated with CHD and the window of vulnerability to cardiac malformation should be set as the weeks 3–8 of pregnancy (fertilization to 8 weeks, most lesions at 3–5 weeks) because this is a sensitive time for embryonic organs formation [18, 23, 44]. Furthermore, major structural cardiac abnormalities can be identified at 11 to 13 weeks of gestation and most pregnant patients have a routine ultrasound for anatomic survey at around 18–20 weeks at gestation [45, 46]. Therefore, we hypothesized that the first trimester, especially 3–8 weeks of pregnancy, is more critical for heart development, and set days 1–56 at gestation (from date of LMP to the end of week 8 at gestation) as lag time instead of the entire pregnancy period. In our results, vulnerable windows of PM_2.5_, O_3_, and SO_2_ were 1^st^–2^nd^ gestation weeks, the 1^st^ gestation week, and 6^th^–7^th^ gestation weeks, respectively. No association was found in PM_10_, CO, and NO_2_ in our study. The difference in concentration of study area, choice of lag time, different outcomes in studies may result in the various conclusions. Our stratified analysis results were inconsistent with previous studies [22, 47], which indicated that baby boys were more sensitive to air pollutants than girls in specific air pollutants or the relationships between air pollutants and CHD were similar to boys and girls. Consistent with Stingone et al. [33], we found that girls may be more sensitive to air pollution. Previous studies indicated that the prevalence of all CHD was consistently higher in females for both adult and pediatric populations and simple CHD subtypes, such as ASD, have shown a female preponderance [48, 49]. Therefore, we consider that in addition to the different prevalence of CHD by infant sex, differences in proportion of CHD subtypes in each study may have contributed to the inconsistent results.

For particulate matter, against PM_2.5_, no association was found between PM_10_ and CHD in this study. It is possible that compared with larger particulate matter, smaller particles show greater deposition in the deep lung and have a high surface area-to-mass ratio, potentially leading to enhanced biological toxicity [50]. It is inferred that structural abnormalities and functional changes in the placenta might have deleterious effects on the development of the fetal heart [52]. Evidence on the presence of air pollution nanoparticles in placental tissue cells provides a mechanism by which this form of pollution could affect the placenta and potentially be transported to the fetus, inducing direct effects [53, 54]. For the exposure to sulfur dioxide, SO_2_ need to be converted to bisulfite and sulfite to disturb the normal development of the embryo by free radicals formed during sulfite oxidation [51]. These various biological pathways may be responded to risks of developing CHD with different lag periods of air pollutants. Study on analysis of inherited and noninherited risk of CHD indicated that environment factors, such as particulate matters, may be involved in the pathogenesis of CHD, whereas the contribution of environmental exposure was unknown [55]. Epidemiological and experimental studies are needed to clarify the mechanism of the air pollution effect on CHD.

Limitations in our study cannot be overlooked. First, our data were derived from post-natal screening programs. The majority of CHD cases were ventricular septal defects and atrial septal defects, which proportions were 36.1% and 62.3% in our study cases, respectively. The survival rates of critical CHDs (such as tetralogy of Fallot, transposition of the great arteries) are lower than noncritical CHDs [56], which would lead some pregnant women to choose induction of labor rather than delivery when the fetus was found to have critical CHDs during pregnancy. It might bring in underestimation of the percentage of CHD in pregnant women. Thus, the association between air pollution and congenital heart disease may be influenced by survival bias. Second, individual exposure estimated by using ambient concentrations of pollutants at their residential location does not account for indoor concentration. Third, individual concentration was calculated by IDW, which cannot capture the variation for participants living close to major roads. However, the potential misclassification bias for the exposure assessment tended to be toward the null, indicating that our results may underestimate the effect strengths of associations. Therefore, we will focus on optimizing individual exposure measurement methods, and subdividing the types of congenital heart disease in future studies.

## 5. Conclusions

In summary, the nested case-control study indicated that the exposure to PM_2.5_, O_3_, and SO_2_ in early stage during pregnancy had lag effects on congenital heart disease. The exposure window mainly concentrates in 1^st^–7^th^ weeks during pregnancy, varying with different types of pollutants. Epidemiologic studies with more detailed exposure assessments are warranted to validate our findings and our results reinforce the need for mitigating air pollution exposure to in the early pregnancy stage, especially since the impact of PM_2.5_ on CHD may precede heart development.

## Abbreviations

CO: Carbon Monoxide
NO_2_: Nitrogen Dioxide
O_3_: ozone
PM_2.5_: fine particulate matter (aerodynamic diameter <2.5 µm)
PM_10_: particulate matter (aerodynamic diameter <10 µm)
SO_2_: sulfur dioxide
CHD: congenital heart disease

## Supplementary information

**Additional file 1:** **eTable 1** Concentration of air pollutants in Tianjin during 2013–2015. **eTable 2** The detailed ORs (95%CI) for different lag days of each air pollutants. **eTable 3** The detailed ORs (95%CI) for different lag days of PM_2.5_, SO_2_, and O_3_ in stratified analysis. **eFigure 1** Correlations between individual exposure concentration of each pollutant. **eFigure 2** Associations of ORs between air pollutants and CHD in sensitivity analysis. **eFigure 3** The ORs for different lag days of each air pollutants in sensitivity analysis.

## Declarations

### Availability of data and material

The data will not available to the others except for researchers with investigator support after the approval of The Ethics Committee for Clinical Research of Tianjin Women and Children’s Health Center.
