# Supplementary material for: Early prenatal exposure to air pollutants and congenital heart disease: a nested case-control study
Source: Environ Health Prev Med. 2023 Jan 13;28:4. doi: 10.1265/ehpm.22-00138 (PMC9845069; doi:10.1265/ehpm.22-00138)
Supplement: Supplementary file 1 — Additional file 1: eTable 1 Concentration of air pollutants in Tianjin during 2013–2015. eTable 2 The detailed ORs (95%CI) for different lag days of each air pollutants. eTable 3 The detailed ORs (95%CI) for different lag days of PM2.5, SO2, and O3 in stratified analysis. eFigure 1 Correlations between individual exposure concentration of each pollutant. eFigure 2 Associations of ORs between air pollutants and CHD in sensitivity analysis. eFigure 3 The ORs for different lag days of each air pollutants in sensitivity analysis. [file ehpm-28-004-s001.docx]

**Supplementary Materials**

**eTable 1** Concentration of air pollutants in Tianjin during 2013-2015.

| Exposure | Range | P_25_ | P_50_ | P_75_ | references |
| --- | --- | --- | --- | --- | --- |
| PM_2.5_（μg/m^3^） | 9.05-374.15 | 42.31 | 66.54 | 102.03 | 75 |
| PM_10_（μg/m^3^） | 11.65-450.99 | 63.29 | 97.14 | 144.69 | 150 |
| CO （mg/m^3^） | 0.18-8.38 | 0.96 | 1.38 | 1.91 | 4 |
| SO_2_ （μg/m^3^） | 1.68-211.47 | 14.50 | 26.01 | 49.96 | 150 |
| NO_2_（μg/m^3^） | 5.96-175.94 | 29.37 | 39.80 | 57.67 | 80 |
| O_3_ （μg/m^3^） | 2.71-168.00 | 25.53 | 46.20 | 72.29 | 160 |

a. The references were 24-hour average concentration of PM_2.5_, PM_10_, CO, SO_2_ and NO_2_; and the 8-hour maximum average concentration of O_3_ in national secondary standard (GB3095-2012).

**eTable 2** The detailed ORs (95%CI) for different lag days of each air pollutants

|  | PM_2.5_ | PM_10_ | CO | O_3_ | NO_2_ | SO_2_ |
| --- | --- | --- | --- | --- | --- | --- |
| lag0 | 1.014（0.998,1.030） | 1.018（0.954,1.086） | 1.175（0.639,2.161） | **1.485（1.070,2.062）** | 1.062（0.865,1.304） | 0.773（0.406,1.468） |
| lag1 | 1.013（0.999,1.028） | 1.016（0.959,1.077） | 1.178（0.676,2.053） | **1.432（1.067,1.923）** | 1.055（0.875,1.271） | 0.763（0.422,1.379） |
| lag2 | 1.013（1.000,1.026） | 1.015（0.963,1.069） | 1.181（0.713,1.957） | **1.382（1.063,1.796）** | 1.047（0.885,1.240） | 0.753（0.436,1.301） |
| lag3 | **1.013（1.001,1.024）** | 1.013（0.967,1.062） | 1.184（0.749,1.874） | **1.333（1.057,1.681）** | 1.040（0.894,1.211） | 0.744（0.448,1.235） |
| lag4 | **1.012（1.002,1.023）** | 1.012（0.971,1.055） | 1.187（0.781,1.805） | **1.287（1.050,1.577）** | 1.034（0.902,1.184） | 0.736（0.458,1.182） |
| lag5 | **1.012（1.002,1.021）** | 1.010（0.974,1.048） | 1.190（0.809,1.751） | **1.243（1.040,1.486）** | 1.027（0.909,1.160） | 0.728（0.464,1.142） |
| lag6 | **1.011（1.003,1.020）** | 1.009（0.977,1.042） | 1.192（0.830,1.713） | **1.202（1.026,1.408）** | 1.020（0.914,1.139） | 0.721（0.467,1.114） |
| lag7 | **1.011（1.003,1.018）** | 1.008（0.979,1.037） | 1.194（0.843,1.690） | **1.163（1.008,1.342）** | 1.014（0.917,1.122） | 0.715（0.466,1.098） |
| lag8 | **1.010（1.003,1.017）** | 1.007（0.980,1.034） | 1.195（0.849,1.684） | 1.127（0.985,1.288） | 1.008（0.917,1.108） | 0.711（0.463,1.092） |
| lag9 | **1.010（1.003,1.017）** | 1.005（0.980,1.031） | 1.196（0.847,1.690） | 1.093（0.958,1.247） | 1.002（0.916,1.098） | 0.708（0.457,1.096） |
| lag10 | **1.009（1.003,1.016）** | 1.004（0.980,1.030） | 1.197（0.839,1.707） | 1.062（0.928,1.215） | 0.997（0.911,1.091） | 0.706（0.450,1.106） |
| lag11 | **1.009（1.002,1.016）** | 1.004（0.978,1.029） | 1.197（0.827,1.731） | 1.033（0.897,1.190） | 0.992（0.905,1.087） | 0.706（0.443,1.123） |
| lag12 | **1.008（1.001,1.016）** | 1.003（0.977,1.030） | 1.196（0.813,1.758） | 1.007（0.866,1.171） | 0.987（0.898,1.086） | 0.707（0.437,1.144） |
| lag13 | 1.008（1.000,1.016） | 1.002（0.975,1.031） | 1.194（0.798,1.786） | 0.984（0.838,1.155） | 0.983（0.891,1.085） | 0.711（0.433,1.168） |
| lag14 | 1.007（0.999,1.015） | 1.002（0.973,1.032） | 1.192（0.783,1.812） | 0.963（0.813,1.141） | 0.979（0.883,1.086） | 0.717（0.430,1.194） |
| lag15 | 1.007（0.999,1.015） | 1.001（0.971,1.033） | 1.188（0.770,1.833） | 0.945（0.791,1.129） | 0.976（0.876,1.087） | 0.725（0.430,1.221） |
| lag16 | 1.006（0.998,1.015） | 1.001（0.970,1.034） | 1.184（0.758,1.848） | 0.929（0.773,1.117） | 0.973（0.871,1.088） | 0.736（0.433,1.248） |
| lag17 | 1.006（0.997,1.015） | 1.001（0.969,1.035） | 1.179（0.749,1.855） | 0.916（0.760,1.105） | 0.971（0.866,1.088） | 0.749（0.440,1.275） |
| lag18 | 1.005（0.997,1.014） | 1.002（0.969,1.035） | 1.172（0.742,1.852） | 0.906（0.750,1.094） | 0.969（0.864,1.087） | 0.766（0.451,1.300） |
| lag19 | 1.005（0.996,1.014） | 1.002（0.970,1.036） | 1.165（0.737,1.841） | 0.898（0.745,1.082） | 0.968（0.863,1.086） | 0.786（0.467,1.325） |
| lag20 | 1.004（0.996,1.013） | 1.003（0.971,1.036） | 1.157（0.735,1.820） | 0.893（0.745,1.072） | 0.967（0.863,1.083） | 0.811（0.487,1.348） |
| lag21 | 1.004（0.996,1.012） | 1.004（0.972,1.036） | 1.147（0.734,1.794） | 0.891（0.747,1.062） | 0.967（0.865,1.081） | 0.838（0.513,1.370） |
| lag22 | 1.003（0.995,1.012） | 1.005（0.975,1.036） | 1.137（0.734,1.763） | 0.890（0.752,1.054） | 0.967（0.868,1.077） | 0.869（0.543,1.392） |
| lag23 | 1.003（0.995,1.011） | 1.006（0.977,1.035） | 1.127（0.734,1.730） | 0.892（0.759,1.047） | 0.967（0.872,1.074） | 0.903（0.577,1.414） |
| lag24 | 1.002（0.995,1.010） | 1.007（0.979,1.035） | 1.116（0.734,1.698） | 0.895（0.767,1.044） | 0.968（0.876,1.070） | 0.941（0.615,1.438） |
| lag25 | 1.002（0.995,1.009） | 1.008（0.982,1.035） | 1.105（0.732,1.667） | 0.900（0.776,1.044） | 0.969（0.881,1.067） | 0.980（0.656,1.464） |
| lag26 | 1.001（0.994,1.009） | 1.010（0.984,1.035） | 1.093（0.729,1.641） | 0.906（0.784,1.049） | 0.970（0.885,1.064） | 1.022（0.699,1.494） |
| lag27 | 1.001（0.994,1.008） | 1.011（0.986,1.036） | 1.082（0.723,1.619） | 0.914（0.790,1.058） | 0.972（0.889,1.062） | 1.066（0.743,1.530） |
| lag28 | 1.000（0.993,1.008） | 1.012（0.988,1.037） | 1.070（0.715,1.602） | 0.923（0.795,1.072） | 0.973（0.892,1.061） | 1.112（0.786,1.573） |
| lag29 | 1.000（0.992,1.008） | 1.013（0.989,1.039） | 1.059（0.705,1.591） | 0.933（0.798,1.090） | 0.974（0.895,1.061） | 1.158（0.826,1.623） |
| lag30 | 1.000（0.992,1.008） | 1.014（0.989,1.040） | 1.048（0.693,1.584） | 0.944（0.801,1.113） | 0.975（0.896,1.061） | 1.205（0.863,1.682） |
| lag31 | 0.999（0.991,1.008） | 1.016（0.989,1.042） | 1.037（0.681,1.581） | 0.955（0.802,1.137） | 0.976（0.897,1.063） | 1.250（0.894,1.748） |
| lag32 | 0.999（0.990,1.008） | 1.016（0.989,1.045） | 1.027（0.668,1.580） | 0.967（0.804,1.164） | 0.977（0.897,1.065） | 1.295（0.921,1.820） |
| lag33 | 0.998（0.989,1.008） | 1.017（0.989,1.047） | 1.017（0.655,1.580） | 0.979（0.806,1.191） | 0.978（0.896,1.067） | 1.336（0.943,1.893） |
| lag34 | 0.998（0.989,1.008） | 1.018（0.988,1.049） | 1.008（0.643,1.580） | 0.992（0.808,1.216） | 0.978（0.895,1.069） | 1.374（0.961,1.965） |
| lag35 | 0.998（0.988,1.008） | 1.018（0.987,1.050） | 1.000（0.634,1.578） | 1.004（0.812,1.240） | 0.977（0.893,1.070） | 1.407（0.975,2.030） |
| lag36 | 0.997（0.987,1.008） | 1.018（0.987,1.051） | 0.993（0.626,1.573） | 1.016（0.818,1.261） | 0.977（0.892,1.069） | 1.434（0.987,2.083） |
| lag37 | 0.997（0.987,1.007） | 1.018（0.986,1.051） | 0.986（0.622,1.563） | 1.027（0.826,1.277） | 0.975（0.891,1.067） | 1.454（0.998,2.120） |
| lag38 | 0.997（0.987,1.007） | 1.018（0.986,1.051） | 0.980（0.620,1.549） | 1.037（0.835,1.289） | 0.974（0.891,1.064） | **1.466（1.007,2.136）** |
| lag39 | 0.997（0.987,1.007） | 1.017（0.985,1.049） | 0.975（0.622,1.531） | 1.047（0.846,1.296） | 0.971（0.891,1.059） | **1.471（1.015,2.132）** |
| lag40 | 0.997（0.987,1.007） | 1.016（0.985,1.048） | 0.971（0.625,1.509） | 1.057（0.859,1.299） | 0.968（0.891,1.052） | **1.469（1.022,2.109）** |
| lag41 | 0.996（0.987,1.006） | 1.015（0.985,1.046） | 0.968（0.631,1.486） | 1.065（0.874,1.298） | 0.965（0.891,1.045） | **1.459（1.029,2.070）** |
| lag42 | 0.996（0.987,1.006） | 1.013（0.984,1.043） | 0.966（0.637,1.463） | 1.073（0.889,1.296） | 0.961（0.891,1.037） | **1.444（1.033,2.018）** |
| lag43 | 0.996（0.987,1.005） | 1.012（0.984,1.041） | 0.964（0.644,1.443） | 1.080（0.903,1.292） | 0.957（0.889,1.030） | **1.423（1.035,1.956）** |
| lag44 | 0.996（0.987,1.005） | 1.010（0.982,1.038） | 0.963（0.650,1.426） | 1.087（0.917,1.290） | 0.952（0.886,1.024） | **1.398（1.034,1.889）** |
| lag45 | 0.996（0.987,1.005） | 1.008（0.980,1.036） | 0.962（0.653,1.416） | 1.094（0.927,1.290） | 0.948（0.881,1.019） | **1.368（1.028,1.821）** |
| lag46 | 0.996（0.987,1.005） | 1.006（0.978,1.035） | 0.961（0.653,1.415） | 1.100（0.934,1.295） | 0.942（0.874,1.017） | **1.335（1.014,1.756）** |
| lag47 | 0.996（0.987,1.005） | 1.004（0.974,1.034） | 0.962（0.649,1.426） | 1.105（0.934,1.307） | 0.937（0.864,1.017） | 1.299（0.992,1.700） |
| lag48 | 0.996（0.987,1.005） | 1.001（0.970,1.033） | 0.962（0.639,1.449） | 1.110（0.929,1.327） | 0.932（0.851,1.019） | 1.261（0.960,1.655） |
| lag49 | 0.996（0.986,1.006） | 0.999（0.965,1.034） | 0.963（0.624,1.487） | 1.115（0.917,1.356） | 0.926（0.837,1.024） | 1.221（0.919,1.623） |
| lag50 | 0.996（0.985,1.007） | 0.996（0.959,1.035） | 0.964（0.604,1.539） | 1.119（0.900,1.392） | 0.920（0.821,1.031） | 1.181（0.868,1.605） |
| lag51 | 0.996（0.984,1.008） | 0.993（0.952,1.037） | 0.966（0.581,1.604） | 1.124（0.880,1.435） | 0.914（0.804,1.039） | 1.140（0.812,1.599） |
| lag52 | 0.996（0.983,1.009） | 0.991（0.945,1.039） | 0.967（0.556,1.684） | 1.128（0.857,1.485） | 0.908（0.786,1.049） | 1.099（0.754,1.602） |
| lag53 | 0.996（0.982,1.010） | 0.988（0.937,1.041） | 0.969（0.529,1.777） | 1.132（0.832,1.540） | 0.902（0.768,1.059） | 1.058（0.694,1.613） |
| lag54 | 0.996（0.981,1.012） | 0.985（0.930,1.044） | 0.971（0.501,1.883） | 1.136（0.806,1.601） | 0.895（0.749,1.070） | 1.019（0.637,1.631） |
| lag55 | 0.996（0.980,1.013） | 0.982（0.922,1.047） | 0.973（0.473,2.001） | 1.139（0.780,1.666） | 0.889（0.731,1.082） | 0.981（0.582,1.653） |

a. The odd ratios (ORs) are shown for 10 μg/m^3^ increase in PM_10_, PM_2.5_, NO_2_, SO_2_, and O_3_, for 1mg/m^3^ increase in CO.

b. Bold values mean the significant ORs（95%CI）

**eTable 3** The detailed ORs (95%CI) for different lag days of PM_2.5_, SO_2_, and O_3_ in stratified analysis.

| Air pollutants | Lag days | Total(n=8748) | Girls(n=5058) | Boys(n=3690) |
| --- | --- | --- | --- | --- |
| PM_2.5_ |  |  |  |  |
|  | lag3 | **1.013（1.001,1.024）** | 1.014（0.998,1.030） | 1.010（0.992,1.028） |
|  | lag4 | **1.012（1.002,1.023）** | 1.014（1.000,1.028） | 1.010（0.994,1.026） |
|  | lag5 | **1.012（1.002,1.021）** | **1.014（1.001,1.026）** | 1.009（0.995,1.023） |
|  | lag6 | **1.011（1.003,1.020）** | **1.013（1.002,1.025）** | 1.008（0.996,1.021） |
|  | lag7 | **1.011（1.003,1.018）** | **1.013（1.003,1.024）** | 1.008（0.997,1.019） |
|  | lag8 | **1.010（1.003,1.017）** | **1.013（1.003,1.023）** | 1.007（0.997,1.018） |
|  | lag9 | **1.010（1.003,1.017）** | **1.012（1.003,1.022）** | 1.007（0.997,1.017） |
|  | lag10 | **1.009（1.003,1.016）** | **1.012（1.003,1.021）** | 1.006（0.996,1.017） |
|  | lag11 | **1.009（1.002,1.016）** | **1.012（1.002,1.021）** | 1.006（0.995,1.016） |
|  | lag12 | **1.008（1.001,1.016）** | **1.011（1.002,1.021）** | 1.005（0.994,1.016） |
|  | lag13 | 1.008（1.000,1.016） | **1.011（1.001,1.021）** | 1.005（0.993,1.017） |
| SO_2_ |  |  |  |  |
|  | lag38 | **1.466（1.007,2.136）** | 1.588（0.953,2.648） | 1.412（0.765,2.608） |
|  | lag39 | **1.471（1.015,2.132）** | 1.629（0.983,2.700） | 1.396（0.762,2.558） |
|  | lag40 | **1.469（1.022,2.109）** | **1.658（1.011,2.720）** | 1.375（0.762,2.481） |
|  | lag41 | **1.459（1.029,2.070）** | **1.676（1.036,2.711）** | 1.350（0.764,2.383） |
|  | lag42 | **1.444（1.033,2.018）** | **1.682（1.057,2.677）** | 1.320（0.768,2.270） |
|  | lag43 | **1.423（1.035,1.956）** | **1.679（1.074,2.624）** | 1.288（0.773,2.148） |
|  | lag44 | **1.398（1.034,1.889）** | **1.666（1.083,2.561）** | 1.253（0.777,2.022） |
|  | lag45 | **1.368（1.028,1.821）** | **1.644（1.083,2.495）** | 1.216（0.778,1.901） |
|  | lag46 | **1.335（1.014,1.756）** | **1.615（1.071,2.434）** | 1.178（0.774,1.791） |
|  | lag47 | 1.299（0.992,1.700） | **1.580（1.046,2.386）** | 1.138（0.762,1.699） |
|  | lag48 | 1.261（0.960,1.655） | **1.539（1.005,2.357）** | 1.097（0.739,1.629） |
| O_3_ |  |  |  |  |
|  | lag0 | **1.485（1.070,2.062）** | 1.405（0.898,2.200) | 1.573（0.967,2.557） |
|  | lag1 | **1.432（1.067,1.923）** | 1.367（0.913,2.045) | 1.507（0.975,2.331） |
|  | lag2 | **1.382（1.063,1.796）** | 1.329（0.927,1.904) | 1.445（0.981,2.129） |
|  | lag3 | **1.333（1.057,1.681）** | 1.293（0.940,1.778) | 1.385（0.985,1.949） |
|  | lag4 | **1.287（1.050,1.577）** | 1.258（0.949,1.667) | 1.329（0.986,1.792） |
|  | lag5 | **1.243（1.040,1.486）** | 1.225（0.955,1.571) | 1.276（0.983,1.656） |
|  | lag6 | **1.202（1.026,1.408）** | 1.194（0.956,1.491) | 1.226（0.975,1.541） |
|  | lag7 | **1.163（1.008,1.342）** | 1.164（0.950,1.426) | 1.179（0.960,1.447） |

a. The odd ratios (ORs) are shown for 10 μg/m^3^ increase in PM_2.5_, SO_2_, and O_3_.
b. Bold values mean the significant ORs（95%CI）

**eFigure 1** Correlations between individual exposure concentration of each pollutant.


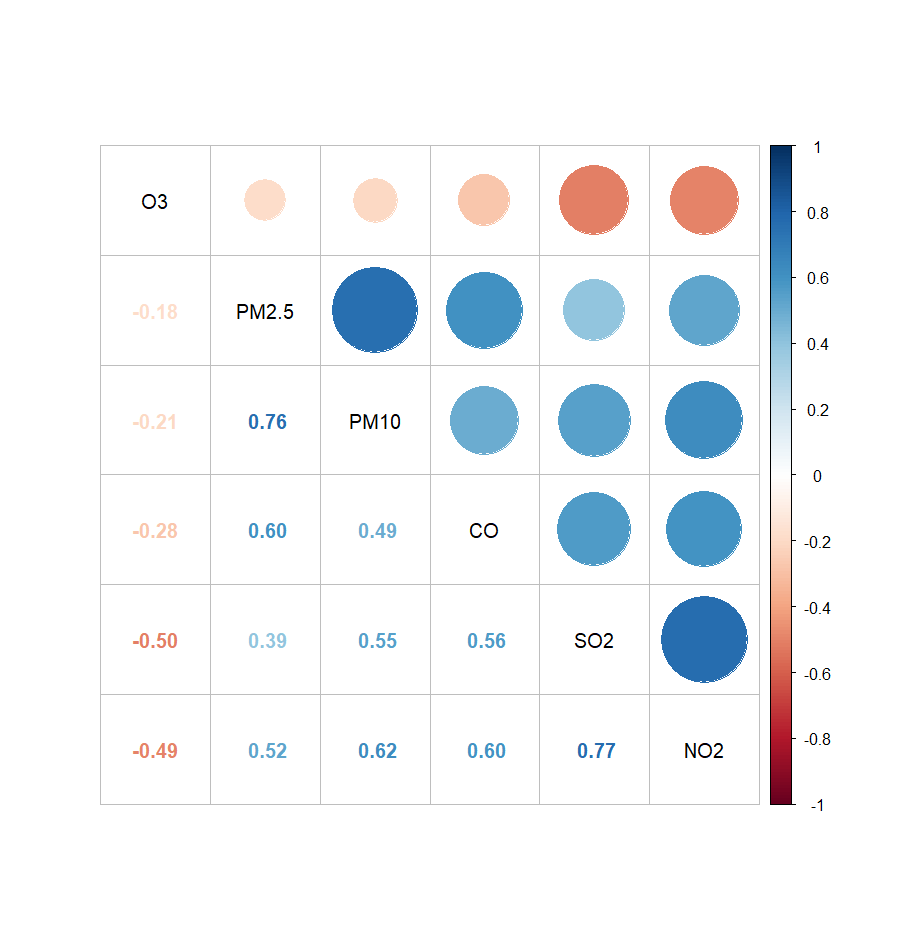


**eFigure 2** Associations of ORs between air pollutants and CHD in sensitivity analysis


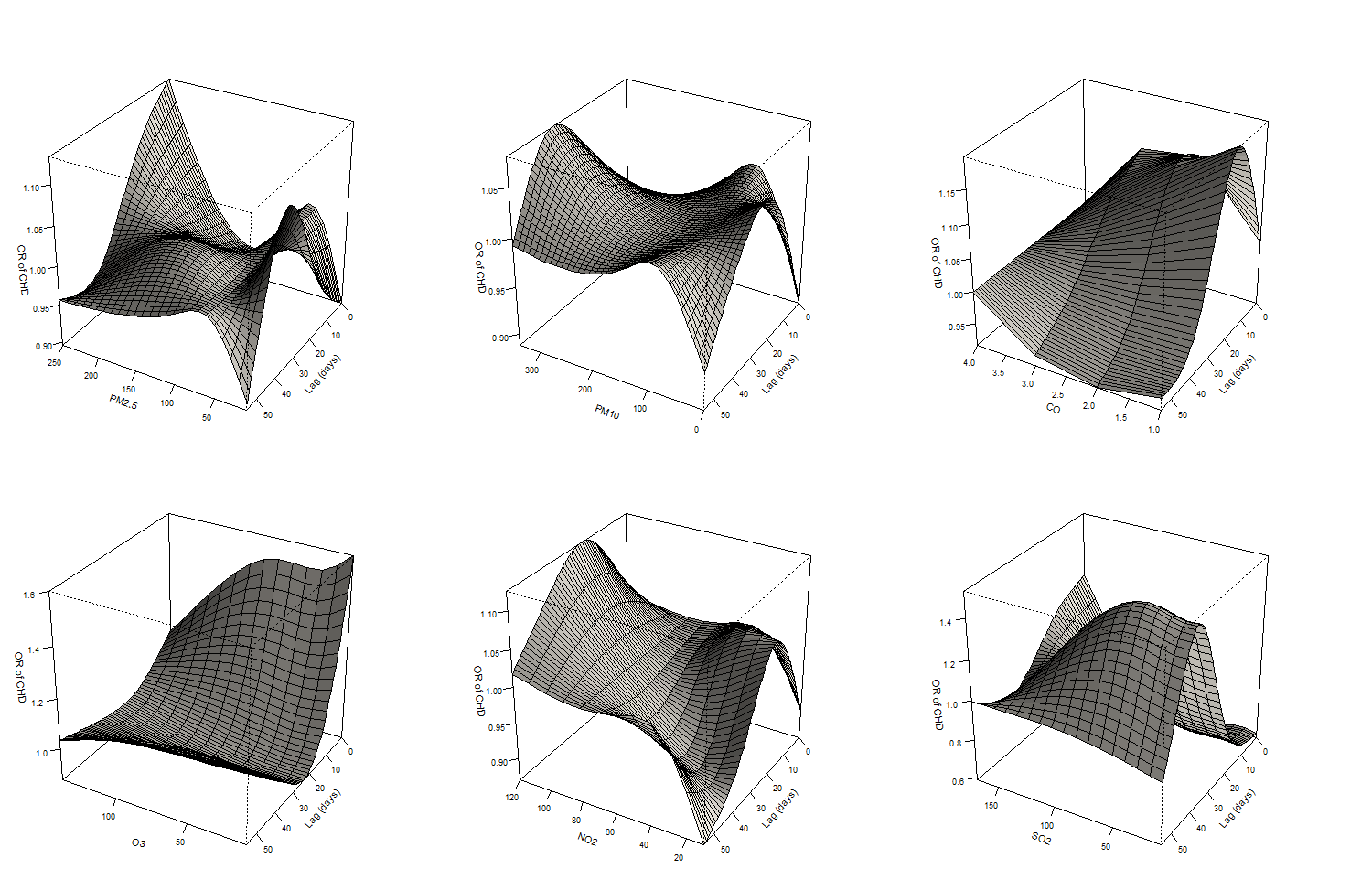


a. 10 μg/m^3^ increase in PM_10_, PM_2.5_, NO_2_, SO_2_, and O_3_; 1mg/m^3^ increase in CO.

**eFigure 3** The ORs for different lag days of each air pollutants in sensitivity analysis.


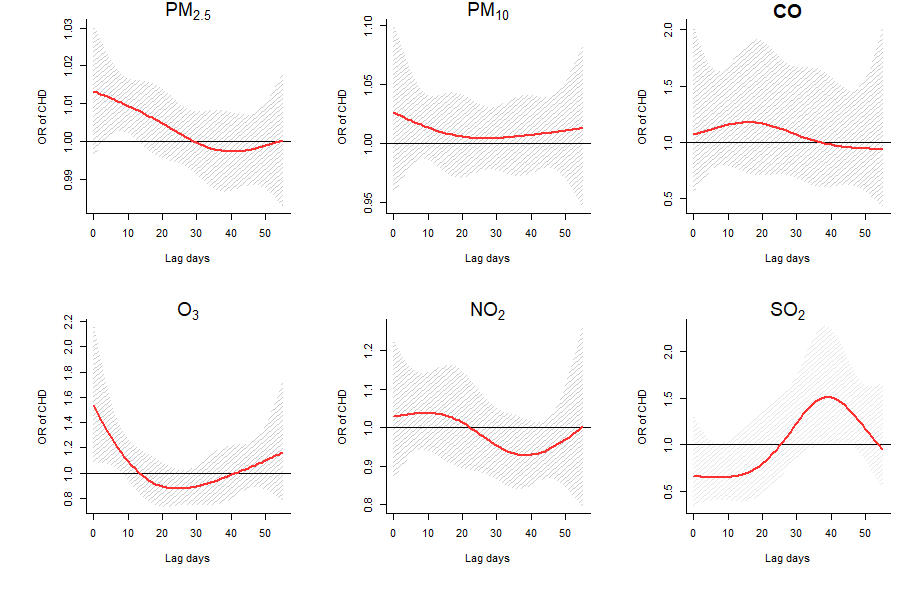


a. 10 μg/m^3^ increase in PM_10_, PM_2.5_, NO_2_, SO_2_, and O_3_; 1mg/m^3^ increase in CO.
